# Genetic and Enzymatic Characteristics of CYP2A13 in Relation to Lung Damage

**DOI:** 10.3390/ijms222212306

**Published:** 2021-11-14

**Authors:** Radim Vrzal

**Affiliations:** Department of Cell Biology and Genetics, Faculty of Science, Palacky University, Slechtitelu 27, 783 71 Olomouc, Czech Republic; radim.vrzal@email.cz; Tel.: +420-58-5634904; Fax: +420-58-5634905

**Keywords:** lung cancer, skatole, NNK, aflatoxin, polymorphism, regulation, FOXA2

## Abstract

Cytochrome P450 2A13 is an omitted brother of CYP2A6 that has an important role in the drug metabolism of liver. Due to extrahepatic expression, it has gained less attention than CYP2A6, despite the fact that it plays a significant role in toxicant-induced pulmonary lesions and, therefore, lung cancer. The purpose of this mini-review is to summarize the basic knowledge about this enzyme in relation to the substrates, inhibitors, genetic polymorphisms, and transcriptional regulation that are known so far (September 2021).

## 1. Introduction

Xenobiotic-metabolizing enzymes (XMEs) play a crucial role in the detoxification of foreign compounds. The most abundant subgroup is the cytochrome P450 superfamily (CYP; 1.14.X.X), a heme that contains enzymes that participate in phase I of biotransformation. Usually, they are bound to the membrane of the endoplasmic reticulum with the C-terminus and using NADPH generated by cytochrome P450 reductase (CYPOR; 1.6.2.4). They incorporate one atom of molecular oxygen into the xenobiotic molecule, and the second one is combined with hydrogen to form water. Due to this property, they are called monooxygenases or mixed function oxidases. The superfamily (CYP) is divided into families (e.g., CYP2) by at least 40% amino-acid sequence homology, and further into subfamilies (e.g., CYP2A), where members must share at least a 55% amino acid identity. Each individual member within the subfamily is further marked with a number (e.g., CYP2A6, CYP2A13). Usually, these are located on the same chromosome, and it is believed that they were created throughout the evolution by gene duplication of the whole *CYP* superfamily [1,2,3]). These enzymes are responsible for the metabolism of xeno-biotics (e.g., drugs, environmental pollutants), as well as endo-biotics (e.g., fatty acids, steroids) [4,5,6]. 

The CYP2A subfamily consists of three complete genes so far: CYP2A6, CYP2A7, and CYP2A13, first identified in 1995 [7]. Due to the primary presence of CYP2A13 outside the gastrointestinal tract, it receives less attention than CYP2A6, which forms approximately 3.5–14% of all human CYPs in the liver [8]. Therefore, it was investigated in more detail towards the drug metabolism. 

Interestingly, CYP2A13 is abundantly expressed in lung tissue and is considered a significant player in the tobacco-induced lung cancer process. One of the key components of cigarette smoke is 4-(methylnitrosamino)-1-(3-pyridyl)-1-butanone (NNK), a tobacco-specific nitrosamine ketone, the metabolism of which is mediated by CYP2A13. Since this compound was labeled as a carcinogen by the International Agency for Research on Cancer (IARC) in 2012, it is inseparably connected with CYP2A13. 

Due to CYP2A13 being less investigated than CYP2A6 (approximately 10 times fewer hits at PUBMED in September 2021), the significant role in the biotransformation of tobacco carcinogens, and possibly the key role in the pathology of tobacco-induced lung cancer, this mini-review summarizes basic knowledge about CYP2A13. 

## 2. Tissue Distribution

The effort to detect CYP2A13 in different human tissues started with the detection of a transcript. The likely reason for this approach was probably the lower detection limit and better control of factors (primer sequence, magnesium concentration) needed for transcript detection. Protein detection was always (and often still is) more challenging; in particular, when there is more than a 93% amino acid sequence similarity with CYP2A6 and the quality of specific antibodies is subjected to an immune system of a given organism, where antibodies are produced. 

Thus, the initial search detected the CYP2A13 transcript in the liver and various extrahepatic tissues (e.g., lung, trachea, brain, mammary gland, prostate, testes) but not in the heart, kidney, bone marrow, colon, small intestine, spleen, stomach, thymus, or skeletal muscle [9]. In this study, the liver tissue contained the least CYP2A13 mRNA, next to the lung < trachea < nasal mucosa. Confirmation of CYP2A13 high expression in olfactory mucosa (OM) was performed in human nasal microsomes from human fetal tissues at different gestational ages (G91-G125) [10]. This study suggested that human fetal OM may be a preferred target tissue for the toxicity of maternally-derived chemical compounds that are activated by the CYP2A enzymes, and may have a greater impact on behavior, growth, and development than in adults.

Other groups have confirmed the relatively high expression of CYP2A13 mRNA in samples of the human lung, bladder, testis, liver and breasts, uterus, and ovary [11], and even in primary human coronary arterial endothelial cells [12]. 

Although transcript detection is relatively straightforward, protein detection caused more confusion. As an example, it may serve the study, where the presence of the CYP2A13 protein was detected in only 12% of 116 human lung microsomal samples by high-resolution immunoblotting followed by immunopurification with an anti-CYP2A5 antibody (mouse ortholog of CYP2A6) [13]. Since the level of CYP2A13, but not CYP2A6, was correlated with lung microsomal NNK metabolic activity, it was speculated that people with relatively high levels of CYP2A13 expression are likely to have an increased risk of developing smoking-related lung cancer. 

A better level of the specific tissue distribution of CYP2A13 was reached with a peptide-specific antibody against human CYP2A13 that did not cross-react with CYP2A6 or CYP2A5 [14]. It was found that a high level of CYP2A13 protein expression can be found in the epithelial cells of the human bronchus and trachea, but a rare distribution in the alveolar cells. Interestingly, there was little expression of the CYP2A13 protein in different types of human lung carcinomas. This suggests that most smoking-related human lung cancers are bronchogenic and that the regulation of CYP2A13 expression is not altered in lung cancer cells [14].

One of the best ways to implicate the role of CYP2A13 in any physiological function is to generate transgenic mice. These were generated using the engineered construct [15] and were found to be normal in morphology, fertility, and development. Furthermore, CYP2A13 was expressed in the respiratory tract, and it was confirmed that it participates in the bioactivation of NNK, a lung procarcinogen.

Unexpectedly, CYP2A13 was found to be expressed in pancreatic α-islet cells by using immunohistochemical analysis (IHC) [16]. Additionally, this enzyme was not expressed in the exocrine portion of the adult human pancreas, nor β-islets. This finding proposed a molecular cause for a significantly elevated risk of pancreatic cancer in current smokers compared to those who had never smoked [17]. 

## 3. Substrates and Inhibitors

CYP2A13 consists of 494 amino acid residues and shares a 93.5% similarity with CYP2A6. Interestingly, they differ in only 32 of their 494 amino acids [18]. The structure of CYP2A13 is composed of 20α-helices and 4β-sheets enclosing the heme group. The active site is very hydrophobic and is approximately 15–20% larger than that of CYP2A6. Amino acids lining the CYP2A13 active site include Phe^107^, Phe^111^, Ala^117^, Phe^118^, Phe^209^, Leu^296^, Asn^297^, Phe^300^, Ala^301^, Glu^304^, Thr^305^, Met^365^, Leu^366^, Leu^370^, and Phe^480^ [18]. Interestingly, only a few of them were repeatedly confirmed in the metabolism of various substrates.

CYP2A13 has a substrate specificity similar to that of CYP2A6, despite the fact that the catalytic efficiency differs. A typical enzymatic activity for the CYP2A subfamily is 7-hydroxylation of coumarin. Other well-known substrates that can be found in tobacco smoke include nicotine, cotinine, and nicotine-derived carcinogen, NNK. Despite the fact that non-consistent results were reported for coumarin [19,20], the catalytic efficiencies between CYP2A13 and CYP2A6 differ for nicotine, cotinine, and NNK. The catalytic efficiency of CYP2A13 is one or two orders of magnitude higher than that of CYP2A6 [21,22]. 

Interestingly, there is not much known about the endogenous substrates of CYP2A13. Based on the homology with mouse orthologs, it was demonstrated that CYP2A13 can metabolize testosterone with Km approx. 13 µM and Vmax approx. 1.7 nmol/min/nmol P450 [23]. Due to the high degree of homology between CYP2A13 and CYP2A6, it can be speculated that some of the endogenous substrates of CYP2A6 can also be metabolized by CYP2A13. However, this must be demonstrated experimentally. 

Tobacco smoke is known to contain thousands of unique compounds; some of them, namely (*R*,*S*)-*N*-nitrosoanatabine (NAT), (*R*,*S*)-*N*-nitrosoanabasine (NAB), and nicotine, inhibited the CYP2A13-mediated metabolism of NNK [24]. Similar inhibitory behavior expressed other two tobacco constituents, namely 1-methyl-4-(3-pyridinyl) pyrrole (beta- nicotyrine) and (-)-menthol, which were found to be CYP2A13 inhibitors with K(i) 0.17 µM and 8.2 µM, respectively [25]. This implies that some tobacco constituents at least partially inhibit carcinogen formation and that there may be considerable variability in the real dose to which a typical smoker is exposed to.

Since the tobacco smoking represents a social habit that is associated with several types of cancer, particularly lung and bladder cancers [26,27,28], it might be helpful to prevent these related consequences in order to decrease the medical and economic burden. The best way would probably be the direct inhibition of tobacco constituents metabolizing biotransformation enzymes, CYPs. Therefore, suitable CYP2A13 inhibitors could help to prevent the toxic consequences of smoking, at least in the lungs.

One of such CYP2A13 inhibitors is 8-methoxypsoralen (8-MOP), which inhibits the metabolism mediated by CYP2A13 of several compounds, including testosterone. The inhibition of CYP2A13 was accompanied by inactivation of the enzyme [23]. The K(I) for the non-competitive inhibition of CYP2A13-mediated coumarin 7-hydroxylation by 8-MOP was 0.11 µM.

Many other compounds of natural origin (e.g., flavonoids apigenin, luteolin, chrysoeriol, quercetin) that can be found in *Vernonia cinerea* and *Pluchea indica* inhibited CYP2A13-mediated coumarin 7-hydroxylation in the sub-micromolar range (Table 1) [29]. Furthermore, this study tested different thiophenes that had significantly lower IC50 values for the pre-incubation mode of the inhibition assay (i.e., reconstituted enzymatic system with tested compounds for 10 min without coumarin). On the contrary, the IC50 was approx. twice as high for the co-incubation mode of inhibition (i.e., the simultaneous presence of tested compounds, reconstituted enzymatic system, and coumarin). 

In another study, rhinacanthins A, B, and C (1,4-naphthoquinone derivatives) (Table 1) isolated from *Rhinacanthus nasutus* potentially and irreversibly inhibited coumarin 7-hydroxylation mediated by purified reconstituted recombinant CYP2A6 and CYP2A13 in a mechanism-based inhibition mode [38]. A similar significant difference between co-incubation and pre-incubation was observed as well. In the search for CYP2A13 inhibitors, some selenium-based compounds were tested for coumarin 7-hydroxylation activity, and their IC50 values were lower for CYP2A13 (0.22–1.4 μM) than for CYP2A6 (2.4–6.2 μM) [37]. These compounds were aromatic selenocyanates (Table 1) that have been shown to prevent cancers caused by PAHs or NNK in experimental animals [41,42,43]. 

The potent inhibitors of CYP2A13 were identified as phenylpropyl isothiocyanate (PPITC) and phenylhexyl isothiocyanate (PHITC), with K(I) values 0.14 and 1.1 uM, respectively [39]. Other tested isothiocynates, such as benzyl isothiocyanate (BITC) and phenethyl isothiocyanate (PEITC), two naturally occurring isothiocyanates, were tested against CYP2A6 and CYP2A13. Interestingly, both compounds displayed lower K(I) towards CYP2A13 than CYP2A6 [40]. This suggested that these isothiocyanates could be developed as chemopreventive agents against lung cancer for smokers who are unwilling or unable to quit smoking. 

The group of potential CYP2A13 inhibitors was expanded to novel synthetic heterocyclic compounds, 1-substituted imidazoles bearing short alkyl chains and possessing high vapor pressure [30]. Here, the IC50 values of some compounds for CYP2A13 were in the low micromolar range, with 1-hexyl-1H imidazole having the lowest value of 2.1 ± 0.1 µM in the coumarin 7-hydroxylation activity assay. 

All of these observations suggest that some CYP2A13 inhibitors could be used directly through the respiratory tract in the form of aerosols before smoking to prevent or slow down cigarette smoke containing pro-carcinogen-induced pulmonary toxicity. However, before applying in men, many variables must be considered, such as the difference between mice and humans, the route of entry (smoking vs. gavage), the dose, or the lack of knowledge about the physiological substrate of CYP2A13. 

However, inhibition might have benefits even for distant organs, such as the bladder, where high expression of CYP2A13 was also found [11]. Since smoking is the predominant risk factor for bladder cancer [17,44], the appropriate inhibition of CYP2A13 might stop the metabolism of some aromatic amines from smoke (e.g., 4-aminobiphenyl) that is metabolized by CYP2A13 into the ultimate carcinogens [11]. 

One of the key aspects in relation to the enzymatic activities are the amino acid residues, usually in the substrate binding pocket. By measuring the spectral binding affinities (K(D)) for nicotine, phenethyl isocyanate (PEITC), coumarin, 2′-methoxyacetophenone (MAP), and 8-MOP, it was demonstrated that the key residues that individually had the largest effect on the CYP2A13 binding of different ligands were residues at positions Ser^208^, Phe^300^, and Ala^301^ [45]. These and three other residues (Ala^213^, Met^365^, Gly^369^) were also found to be important for phenacetin metabolism in CYP2A13 [46]. 

A large group of environmental pollutants is represented by polycyclic aromatic hydrocarbons (PAH), which are, in general, known to be mostly metabolized by CYP1A1/1B1. Interestingly, some research groups found that certain PAHs interacted with and were metabolized by CYP2A13. The interaction that produced type I binding spectra was noted for acenaphthene, acenaphthylene, benzo[c]phenanthrene, fluoranthene, fluoranthene-2,3-diol, and 1-nitropyrene [47]. Particular activity was observed for the activation of 2-aminofluorene and 2-aminoanthracene. Interestingly, a lot of PAHs tested in this study demonstrated IC50 for coumarin 7-hydroxylation activity in the low micromolar range (0.4–10 µM). The ability of CYP2A13 to oxidize naphthalene, phenanthrene, and biphenyl to 1-naphthol, 9-hydroxyphenanthrene, and 2- and/or 4-hydroxybiphenyl, respectively, at much higher rates than CYP2A6, was observed [31]. A detection of mono- and di-oxygenated products produced by CYP2A13 was recorded for pyrene, 1-hydroxypyrene, 1-nitropyrene, and 1-acetylpyrene [32]. Additionally, this study determined the active amino acid residues that are important in directing the orientation of pyrene derivatives at the active sites of CYP2A13 as Ala^301^, Asn^297^, and Ala^117^ [32]. 

The ability of CYP2A13 to convert known or less known compounds was demonstrated for many other compounds, such as 5-hydroxymethyfurfural (5-HMF), which can be found in extracts of cigarettes smoke [33], flavones and flavanones [48], scoparone, a natural bioactive compound found in Chinese herbal medicines [34], naphthalene [49], or N-isobutyldodeca-2E, 4E, 8Z, 10Z-tetraenamide, one of the main component of Echinacea extracts [50]. 

The three most studied/relevant CYP2A13-metabolized compounds are probably aflatoxin B1 (AFB1), NNK, and 3-methylindole (3MI; skatole). 

### 3.1. AFB1

Aflatoxin B1 is a mycotoxin produced by *Aspergillus flavus* and *Aspergillus parasiticus* that is recognized as a potent human and animal liver carcinogen [51]. Next to the ingestion of contaminated foods, humans can be occupationally exposed through contaminated grain dusts, which is associated with respiratory cancer [52,53,54]. 

In 2006, there was the first report that demonstrated the ability of CYP2A13 to efficiently activate the known hepatocarcinogen AFB1 to mutagenic epoxide that was not recorded for CYP2A6 [35]. The important amino acid residues in AFB1 epoxidation and its related toxicity were identified as Ala^117^ and His^372^. The following studies used normal human bronchial epithelial cells (BEAS-2B) that stably expressed CYP2A13 (B-2A13) to demonstrate the involvement of CYP2A13 in AFB1-induced toxicity. The use of this cell line is essential in relation to both the detected high protein expression of CYP2A13 in the human trachea and bronchus and the rare presence in alveolar cells [14]. 

AFB1 dose- and time-dependently induced DNA damage in B-2A13 cells in the concentration range of 5–80 nM [55]. Compared to B-2A6 or B-1A2 cells (stably expressing CYP2A6 or 1A2), B-2A13 cells showed more sensitivity in AFB1-induced phosphorylated histone H2AX (γH2AX) expression, DNA-adduct 8-hydroxy-deoxyguanosine (8-OHdG) formation, and S-phase cell cycle arrest. Furthermore, AFB1 activated the proteins related to DNA damage responses, such as ATM, ATR, Chk2, p53, BRCA1, and H2AX, rather than proteins related to DNA repair. These effects were almost completely inhibited by 100 μM nicotine or 1 μM 8-MOP, CYP2A13 activity inhibitors. Interestingly, the same research group exposed B-2A13 cells to 0.1 nM AFB1, which induced neoplastic transformation and tumor formation in nude mice in passage 30. However, this has occurred in passage 50 in cells stably expressing CYP1A2 [56]. As expected, cells stably expressing CYP2A6 did not develop neoplastic formation. Additionally, AFB1-DNA adducts and 8-OHdG significantly increased in B-2A13 cells in parallel with the upregulation of phosphorylated ATR or BRCA1. 

Due to the structural similarity, aflatoxin G1 (AFG1) was studied in B-2A13 cells as well [57]. Analogically to AFB1, AFG1 increased 8-OHdG and γH2AX in the nucleus and induced S phase arrest and DNA damage in B-2A13 cells. Additionally, proteins related to DNA damage responses, such as ATM, ATR, Chk2, p53, BRCA1, and γH2AX, were activated. Furthermore, all of these effects were inhibited by nicotine or 8-MOP, confirming CYP2A13-mediated AFG1-induced cytotoxicity and DNA damage [57]. 

### 3.2. NNK

4-(methylnitrosamino)-1-(3-pyridyl)-1-butanone is probably the most studied CYP2A13-metabolized compound, due to its pulmonotoxic action. The in vivo proof of concept came in 2014, when CYP2A13-humanized mice (Cyp2A5-null) were used to demonstrate the formation of NNK into a carcinogen [58]. In this study, the levels of O(6)-methylguanine DNA adducts were highly correlated with lung tumorigenesis and were significantly higher in the lungs of CYP2A13-humanized mice than in Cyp2a5-null mice. Interestingly, the magnitude of the differences in lung tumor incidence was greater at low (30 mg/kg) than at high (200 mg/kg) doses of NNK. These results indicated that CYP2A13 is a low Km enzyme in catalyzing NNK bioactivation in vivo and supported the notion that genetic polymorphisms of CYP2A13 can influence the risk of tobacco-induced lung tumorigenesis in humans [58]. The same research group further investigated whether disease-associated inflammation may be responsible for the dramatic variation in CYP2A13 between biopsy samples. CYP2A13-humanized mice had multiple lung tumors at 16 weeks after exposure to NNK (30 or 50 mg/kg) [59]. Interestingly, whereas CYP2A13 mRNA and protein were significantly reduced in NNK-treated groups, pro-inflammatory cytokines, such as TNFα, IFN-γ and IL-6, were significantly higher in the tumor-bearing mice. This indicated lung inflammation at the time of necropsy and a suppression of CYP2A13 levels by inflammation.

An in vitro confirmation of the mutagenic activity of NNK was observed in human lung adenocarcinoma epithelial cells (A549) by the detection of γH2AX [60]. NNK dose-dependently induced γH2AX and induction was suppressed by ATM kinase inhibitors. Interestingly, CYP2A13-overexpressing cells showed a prolonged induction of γH2AX. 

### 3.3. Skatole

In 2007, by investigating the structural differences between CYP2A13 and CYP2A6, the group of Smith et al. discovered the presence of indole in the active site of CYP2A13 [18]. The authors suggested that it was the result of *E. coli*-expressed CYP2A13 and that indole co-purified with protein and constituted the substrate in the active site. Since CYP2A6 has been reported to metabolize indole into various indigoid pigments [61], and due to the similar substrates specificity, it may not be surprising that another pneumotoxicant, skatole (3-methylindole; 3MI), was found to be metabolized by CY2A13 [62]. Physiologically, skatole is produced from tryptophan (Trp) by the gut microbiota, and its importance for lung health lies in the fact that it is formed by the pyrolysis of Trp during tobacco burning. It can be found in cigarette smoke at concentrations ranging from 0.4 to 1.7 µg per cigarette, and these values are higher than for benzo(a)pyrene (BaP) or NNK [63]. These represent two other prototypical carcinogens of cigarette smoke associated with lung tumorigenesis. CYP2A13 dehydrogenates 3MI to 3-methyleneindolenine (3MEIN) and oxygenates 3MI to indole-3-carbinol (I-3-C) and 3-methyloxindole (MOI) [36]. The kinetic parameters for their formation were: V_MAX_ approximately 1.5–1.9 nmol/min/nmol of P450 and K_M_ was 15 and 14 µM, respectively. Additionally, 3MI was found to be a mechanism-based inactivator of CYP2A13, as it produced a loss of activity towards NNK metabolism [36]. The mutagenic potential of 3MI bioactivation by CYP2A13 was further confirmed in 2010, when a significantly higher number of revertants was identified in the *Salmonella typhymurium* strain TA98 (known to detect the most common subtype of cigarette smoke-induced mutagenicity, frameshift mutations in DNA) for 3MI than for BaP and NNK using human lung microsomes [62]. Interestingly, neither human or rat liver S9 subcellular fraction or the use of *S. typhymurium* strain TA100 (detecting base pair substitution mutants) formed mutagenic intermediates of 3MI. In the TA100 strain, only BaP was found to be mutagenic. The use of aminobenzotriazole (ABT), a CYP inhibitor, demonstrated the need for CYP-mediated metabolism to cause mutagenicity. Furthermore, the inhibition of CYP2A13 by 8-MOP decreased the mutagenicity of 3MI and NKK, but not BaP. 

## 4. Genetic Polymorphisms

The *CYP2A* genes can be found on the long arm of chromosome 19 in humans and 7 in mice. They are part of a gene cluster containing several functional *CYP* genes that encode several functional cytochrome P450 enzymes (CYP2A6, CYP2A13, CYP2B6, CYP2F1, and CYP2S1), as well as several CYP pseudogenes [64]. *CYP2A13* is located approx. 70 kbp downstream of *CYP2B6* and immediately upstream of *CYP2F1*. It is well-known that CYP polymorphisms play a significant role in the metabolism of some drugs and pollutants, which may also be important for *CYP2A13.* Since marked interethnic variations in the frequencies of genetic polymorphisms *CYP2A13* were found between French Caucasian, Gabonese, and Tunisian populations [65], it is important to perform subsequent studies that would clearly demonstrate an association between *CYP2A13* haplotypes and the incidence of smoking-related tumors with respect to ethnicity. The *CYP2A13* gene is polymorphic, with more than 20 haplotypes identified to date (https://www.pharmvar.org/gene/CYP2A13; accessed on 28 September 2021). Many of these polymorphisms were characterized toward kinetic parameters [66,67] by using either nicotine or coumarin as substrates. All of these variants displayed reduced catalytic activity. 

Therefore, it is of interest to monitor whether there is a correlation between certain *CYP2A13* polymorphisms and the incidence of any type of cancer. Interestingly, a significant genotype effect was found for the CYP2A13*3 allele and 10 cigarettes smoked per day group within a cohort of Spanish smokers [68]. In contrast, no association between *CYP2A13* polymorphisms and lung cancer was found in the Japanese population [69]. However, the risk of bladder cancer was revealed recently for CYP2A13*1/*2 genotypes in Japanese smokers [70]. 

Another type of association of *CYP2A13* polymorphism and head and neck cancer was observed in a cohort of North Indians [71]. In a comparison of a group of 203 head and neck cancer patients, next to the 201 healthy controls, two novel polymorphisms of *CYP2A13* (T478C and T494C) were detected that were associated with a significantly reduced risk of cancer. On the contrary, the C578T mutant allele of *CYP2A13* was found only in cancer patients. 

In general, most of the genetic polymorphisms for *CYP2A13* are associated with decreased catalytic activity. This has been described for CYP2A13*2 (R257C) and CYP2A13*8 (D158E) towards coumarin 7-hydroxylation, and three variants, R257C, D158E, and CYP2A13*9 (V323L), had two- to threefold decreased catalytic efficiency for NNK hydroxylation [72]. Among the *CYP2A13* allelic variants, the CYP2A13*4 (R101Q) variant was found to be a null enzyme in metabolizing NNK, AFB1, and 5-methoxypsoralen [73]. This variant did not show activity towards coumarin 4-hydroxylation. 

The reduction in lung adenocarcinoma has been associated with CYP2A13*2 genetic polymorphism [74] given by C→T transition, leading to Arg257Cys substitution and reduced activity of *CYP2A13* towards NNK. A stratification analysis demonstrated that the reduced risk of lung adenocarcinoma was related to the *CYP2A13* variant and was limited to smokers, especially light smokers, but not non-smokers or heavy smokers [74]. 

A variant allele of *CYP2A13* (CYP2A13*2) was previously found to be associated with a lower incidence of lung adenocarcinoma in smokers and was associated with a lower level of expression compared to the CYP2A13*1 allele [75]. Furthermore, a 26 nucleotide deletion was discovered, which caused a decrease in *CYP2A13* promoter activity in the human lung cell line A549. These findings suggested that the reported association of the CYP2A13*2 allele with a lower incidence of lung adenocarcinoma in smokers may be at least partially explained by a decrease in CYP2A13 function.

Interestingly, women seem to be prone to an increased risk of early-onset lung cancer if they are carriers of the minor allele of *CYP1B1* SNP rs1056836 (10042C>G/T), but a nonsignificant increased risk was observed for the carriers of the minor allele of *CYP2A13* SNP rs1709084 (13103A>G) [76]. Thus, it was suggested that *CYP1B1* and *CYP2A13* genotypes may contribute to individual susceptibility to early-onset lung cancer in women.

## 5. Transcription Regulation

**Figure 1:**
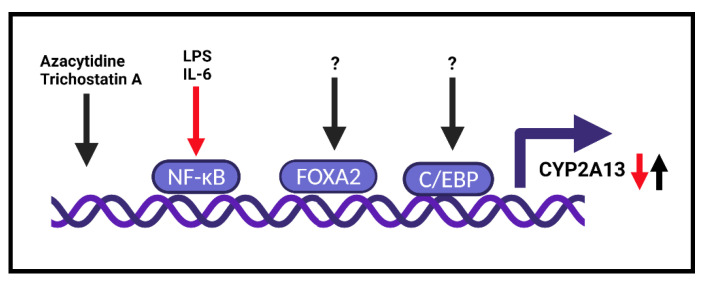
Schematic representation of known transcription regulation of the *CYP2A13* gene.: definition. Red arrow connects the tratment process and the result, i.e. downregualtion. Black arrows connect certain treatments/unknown factors the result of which is the CYP2A13 induction.

The transcription regulation of CYP2A13 is largely unknown, and the main findings are summarized in Figure 1. The expression of CYP2A13 was also found to be affected by different SNPs in the promoter region of CYP2A13. SNPs at -1479T>C, -3101T>G, and -7756G>A possess a suppressive effect in terms of CYP2A13 expression [77]. Furthermore, the negative effect at -1479T>C was enhanced by methylation. 

Some aspects of CYP2A13 expression regulation can be deduced from cancer cells. In the study, where CYP2A13 expression was examined in non-small cell lung cancer (NSCLC) tissues, researchers observed the downregulation of CYP2A13 in lung adenocarcinoma [78]. This observation was confirmed in another study [79]. On the contrary, a marked increase in CYP2A13 in NSCLC was described in 2010 using immunohistochemical analysis [80]. However, it was suggested that the high expression of CYP2A13 might be associated with tumor development and progression in non-small cell lung carcinomas.

Thus, CYP2A13 expression seems to be affected at the transcription level by transcription factors that reflect the surrounding environment. One such factor is the CCAAT/enhancer binding protein (C/EBP) that was found to regulate human CYP2A13 [81]. Two C/EBP binding elements were identified with the use of a reporter gene assay with a 216 bp promoter fragment of CYP2A13 together with the chromatin immunoprecipitation (ChIP) assay. Thus, the activation and binding of C/EBP to the promoter of CYP2A13 was confirmed (Figure 1). 

One of the transcription factors that was found to be involved in the regulation of CYP2A13 was FOXA2, a forkhead box A, also known as hepatocyte nuclear factor 3beta (HNF-3B). It is a critical regulator of airway epithelial differentiation and lung development, where it regulates genes that mediate surfactant homeostasis and host defense, both required in postnatal adaptation for air breathing [82]. When performing an in silico scan of binding sites followed by ChIP, it was found that FOXA2 binds to the CYP2A13 promoter [83] (Figure 1). 

The possible regulation or implication of the estrogen receptor (ER) in CYP2A13 expression can be speculated from the case control study, where a nonsignificant increase in lung cancer risk was observed in the group of women carriers of the minor allele of *CYP2A13* SNP rs1709084 (13103A>G), in which, the effect was further modified by smoking [76]. In particular, for light and never-smokers (0–10 pack-year), this minor genotype was associated with an increased risk of lung cancer. Furthermore, the three-way interaction between gender, smoking, and the genotype of *CYP2A13* was statistically significant [76]. It was concluded that this *CYP2A13* genotype may contribute to individual susceptibility to early-onset lung cancer in women. However, so far, that would investigate or identify the relationship between ER and/or FOXA2 and CYP2A13 expression, there are some indications that such a relationship could exist. One such indicia comes from studies that tried to decipher sexual dimorphism for hepatocellular carcinoma (HCC), where a significantly lower incidence in women exists [84]. Since this phenomenon is the same for rodents as it is for humans, it was demonstrated in female mouse liver that Foxa2 is complexed with ERalpha [85]. However, since these observations were made in mice and in liver, not in humans and in the lungs or bladder, the true role of ER in CYP2A13 expression represents a further research direction. Similarly, the very same role of the androgen receptor (AR) should also be investigated, since a direct interaction of AR with Foxa2 was identified in the mouse epididymal epithelial cell line DC2 [86]. Therefore, sexual hormones can affect the expression of CYP2A13, but this must be verified experimentally.

The expression level of some CYPs is downregulated by inflammation. Similar effects seem to apply to CYP2A13 as well. At the molecular level, this was confirmed in 2013 in a human lung cell line, NCI-H441 [87]. Exposure of NCI-H441 cells to lipopolysaccharide (LPS) and proinflammatory cytokine (IL-6) suppressed CYP2A13 mRNA expression (Figure 1). Similarly, the administration of an intraperitoneal injection of LPS into CYP2A13-humanized mice resulted in systemic and lung inflammation that substantially reduced CYP2A13 mRNA and protein levels. The authors found one major (between -484 and -1008 bp) and other minor (between -134 and -216 bp) critical regions in the CYP2A13 promoter. The major one was identified as NF-kB binding sites by the gel shift assay and the reporter gene assay [87]. Furthermore, the addition of pyrrolidine dithiocarbamate (PDTC), a NF-kB inhibitor, prevented the LPS-mediated suppression of CYP2A13. This study confirmed that inflammation leads to the suppression of CYP2A13 expression levels and contributed to the large differences between individuals in CYP2A13 levels previously found in human lung biopsy samples [13]. 

However, the role of inflammation can be tricky, since AFG1, which can induce chronic lung inflammation, increased CYP2A13 expression together with DNA damage in AFG1-induced inflamed lung tissues [88]. Furthermore, mice treated with the soluble TNFα receptor and AFG1 demonstrated that TNFα inhibited AFG1-induced chronic lung inflammation in vivo and reversed CYP2A13 expression and DNA damage in primary mouse alveolar type II (AT-II) cells. However, by using human AT-II-like cells (A549), the authors found an enhancement of AFG1-induced DNA damage with TNFα. Blocking the NF-kB pathway with siRNA resulted in the inhibition of TNFα-induced CYP2A13 [88]. 

One of the factors that contributes to different expression is represented by epigenetic mechanisms, such as methylation status. This was studied for the CYP2A13 gene in head and neck cancer tissues. Aberrant hypermethylation of 27.4% was observed in cancer tissue, along with healthy tissue, at 15.8% [89]. A significant interaction between smoking and the methylation status of CYP2A13 was observed, and it was suggested that the hypermethylation of CYP2A13 is independent from and in interaction with a smoking associated with an increased risk of head and neck cancer. 

Interestingly, by using epigenetic agents (azacytidine—DNA demethylation agent and trichostatin A—histone deacetylation inhibitor), it was found that CYP2A13 expression is induced in NCI-H441 human lung cancer cells [81] (Figure 1). This observation was confirmed 6 years later, and the presence of LPS or IL-6 suppressed the induction of CYP2A13 mRNA by epigenetic agents [87].

## 6. Conclusions

Cytochrome P450 2A13 appears to be an important player in certain toxicants-induced lung inflammation, and, consequently, is at least partially responsible for lung tumorigenesis. Therefore, a comprehensive understanding of the exact distribution inside the lungs, the level of expression, and the regulation by different transcription factors may help to aim this molecular target towards selective medical interventions. These may include the suppression of inflammation or direct CYP2A13 enzymatic inhibition. Furthermore, since CYP2A13 is not restricted to only lung tissue, a preventive measure can also be used for other organs, such as the bladder or pancreas. However, this would probably include a more complex medical strategy. Additionally, the abundance of CYP2A13 expression in the lung tissue likely reflects the physiological need and identification of a physiological substrate, which would help to better understand the role of CYP2A13 in the lungs’ physiology. Another point of interest may represent an intestine microbiota-formed skatole that can absorb into systemic circulation. Although plasma levels are very low and mostly undetectable, an important question arises regarding whether chronic exposure to these nano- or picomolar concentrations of skatole for two to four decades of human life could be associated with non-smoker-induced lung cancer. This and other questions require further research.

## Figures and Tables

**Table 1:** Selected substrates and inhibitors of CYP2A13.

Compound	Relation to CYP2A13	Parameter	Reference
Coumarin	Substrate	Km = 2.21 ± 0.63 or 0.48 ± 0.07 µM, Vmax = 0.69 ± 0.16 or 0.15 ± 0.006	[19,30]
Testosterone	Substrate	Km = 13 ± 3 µM, Vmax = 1.7 ± 0.11	[23]
4-(methylnitrosamino)-1-(3-pyridyl)-1-butanone (NNK)	Substrate	Km = 10.4 µM, Vmax = 3.6	[21]
Nicotine	Substrate/Inhibitor #	Km = 20.2 µM, Vmax = 8.7, Ki = 6.57−25.01 µM ##	[21,24]
Cotinine	Substrate	Km = 45.2 µM, Vmax = 0.7	[21]
4-aminobiphenyl	Substrate	Km = 38.5 ± 0.6 µM, Vmax = 7.8 ± 0.00	[11]
Naphthalene	Substrate	Km = N.D., Vmax = 6.1 ± 0.88	[31]
Phenanthrene	Substrate	Km = N.D., Vmax = 3.14 ± 0.35	[31]
Biphenyl	Substrate	Km = N.D., Vmax = 3.1 ± 0.19	[31]
Pyrene	Substrate	Km = 1.2 ± 0.2 µM, Vmax = 2.0 ± 0.06	[32]
5-hydroxymethyfurfural	Substrate	Km = 50.9 ± 8.3 µM, Vmax = 2.7 ± 0.2	[33]
Scoparone	Substrate	Km = 10.1 µM, Vmax = 22 µmol/min/g	[34]
Aflatoxin B1	Substrate	Km = N.D., Vmax = 1.7–6.2 ##	[35]
3-methylindole (skatole)	Substrate/Inhibitor #	Km = 14.3−14.8 µM, Vmax = 1.5−1.9 ## Ki = 10 µM	[36]
(*R*,*S*)-*N*-nitrosoanatabine (NAT)	Inhibitor #	Ki = 0.21−0.71 µM ##	[24]
(*R*,*S*)-*N*-nitrosoanabasine (NAB)	Inhibitor #	Ki = 0.23−0.87 µM ##	[24]
1-methyl-4-(3-pyridinyl) pyrrole (beta-nicotyrine)	Inhibitor *	Ki = 0.17 µM	[25]
Menthofuran	Inhibitor *	Ki = 1.24 µM	[25]
(-)-menthol	Inhibitor *	Ki = 8.2 µM	[25]
8-methoxypsoralen (8-MOP)	Inhibitor *	Ki = 0.11 µM	[23]
Benzyl selenocyanate (BSC)	Inhibitor *	IC50 = 1.2 ± 0.19 µM	[37]
1,2-phenylenebis(methylene)selenocyanate (o-XSC)	Inhibitor *	IC50 = 1.2 ± 0.13 µM	[37]
1,3-phenylenebis(methylene)selenocyanate (m-XSC)	Inhibitor *	IC50 = 0.22 ± 0.03 µM	[37]
1,4-phenylenebis(methylene)selenocyanate (p-XSC)	Inhibitor *	IC50 = 1.4 ± 0.21 µM	[37]
Apigenin	Inhibitor *	IC50 = 0.05 ± 0.01 µM	[29]
Luteolin	Inhibitor *	IC50 = 0.18 ± 0.02 µM	[29]
Chrysoeriol	Inhibitor *	IC50 = 0.82 ± 0.05 µM	[29]
Quercetin	Inhibitor *	IC50 = 0.80 ± 0.01 µM	[29]
2-(penta-1,3-diyn-1-yl)-5-(4-acetoxy-3-hydroxybuta-1-yn-1-yl) thiophene	Inhibitor *	IC50 = 6.18 ± 0.28 µM	[29]
2-(prop-1-inyl)-5-(6-acetoxy-5-hydroxyhexa-1,3-diinyl) thiophene	Inhibitor *	IC50 = 2.94 ± 0.01 µM	[29]
2-(prop-1-inyl)-5-(5, 6-dihydroxyhexa-1,3-diinyl) thiophene	Inhibitor *	IC50 = 2.40 ± 0.33 µM	[29]
Rhinacanthin-A	Inhibitor *	IC50 = 1.42 ± 0.05 µM	[38]
Rhinacanthin-B	Inhibitor *	IC50 = 1.58 ± 0.17 µM	[38]
Rhinacanthin-C	Inhibitor *	IC50 = 7.1 ± 0.81 µM	[38]
Rhinacanthin-H/I	Inhibitor *	IC50 = 6.5 ± 1.4 µM	[38]
Phenylpropyl isothiocyanate (PPITC)	Inhibitor *	Ki = 0.14 µM	[39]
Phenylhexyl isothiocyanate (PHITC)	Inhibitor *	Ki = 1.1 µM	[39]
1-hexyl-1H imidazole B	Inhibitor *	IC50 = 2.1 ± 0.1 µM	[30]
Benzyl isothiocyanate (BITC)	Inhibitor *	Ki = 1.3 µM	[40]
Phenethyl isothiocyanate (PEITC)	Inhibitor *	Ki = 0.03 µM	[40]

Explanatory notes: *—inhibition considered towards coumarin 7-hydroxylation; #—inhibition considered towards NNK metabolism; ##—for different products formation; N.D.—not determined (unknown); Vmax—the unit in nmol/min/nmol unless specified; Km—Michaelis–Menten constant; Ki—inhibitory constant.

## Abbreviations

3MI	3-methylindole
8-MOP	8-methoxypsoralen
AFB1	aflatoxin B1
AFG1	aflatoxin G1
ATM	ATM serine/threonine kinase
ATR	ATR serine/threonine kinase
BRCA1	Breast cancer type 1 susceptibility protein
ChIP	Chromatin immunoprecipitation
Chk2	Checkpoint kinase 2
FOXA2	Forkhead box A
γH2AX	Phosphorylated H2A.X variant histone
Km	Michaelis–Menten constant
NF-kB	Nuclear factor kappa-light-chain-enhancer of activated B cells
NNK	4-(methylnitrosamino)-1-(3-pyridyl)-1-butanone
p53	tumor protein P53
TNFα	Tumor necrosis factor alpha
Vmax	maximal velocity
